# Quadri-Wave Lateral Shearing Interferogram Outpainting Using QW-GAN for Accurate Wavefront Reconstruction

**DOI:** 10.3390/s26185771

**Published:** 2026-09-11

**Authors:** Yao Fan, Yaxuan Duan, Yiwen Zhang, Zhengshang Da, Yang Yue

**Affiliations:** 1 Advanced Optical Instrument Laboratory, Xi’an Institute of Optics and Precision Mechanics, Chinese Academy of Sciences, Xi’an 710119, China; fanyao@opt.ac.cn (Y.F.); dazhengshang@opt.ac.cn (Z.D.); 2 School of Information and Communications Engineering, Xi’an Jiaotong University, Xi’an 710049, China; yueyang@xjtu.edu.cn; 3 University of Chinese Academy of Sciences, Xi’an 710119, China; 4 Xi’an Key Laboratory of High Power Laser Measurement Technology and Instrument, Xi’an 710119, China; 5 Drilling & Production Technology Research Institute, Chuanqing Drilling Engineering Company Limited, Guanghan 618300, China; zyw19960622@163.com

**Keywords:** quadri-wave generative adversarial networks (QW-GAN), interferogram outpainting, wavefront reconstruction, gibbs phenomenon mitigation

## Abstract

This paper proposes a novel outpainting technique for quadri-wave lateral shearing interferograms which employs Quadri-Wave Generative Adversarial Networks (QW-GANs). QW-GAN is adversarially trained to capture the high-order statistics of real interferograms. It thereby avoids the Gibbs-ringing artifacts and spectral leakage caused by deterministic interpolation or single-frame extrapolation. The recovered fringes remain physically consistent with preserved Fourier support, enabling more accurate wavefront phase retrieval. Numerical simulations and comparisons with aberrations (defocus, astigmatism, coma, spherical, and random), along with real experimental results, demonstrate the enhanced reconstruction precision of our method, providing an efficient solution for wavefront reconstruction in optical applications.

## 1. Introduction

**Figure 1:**
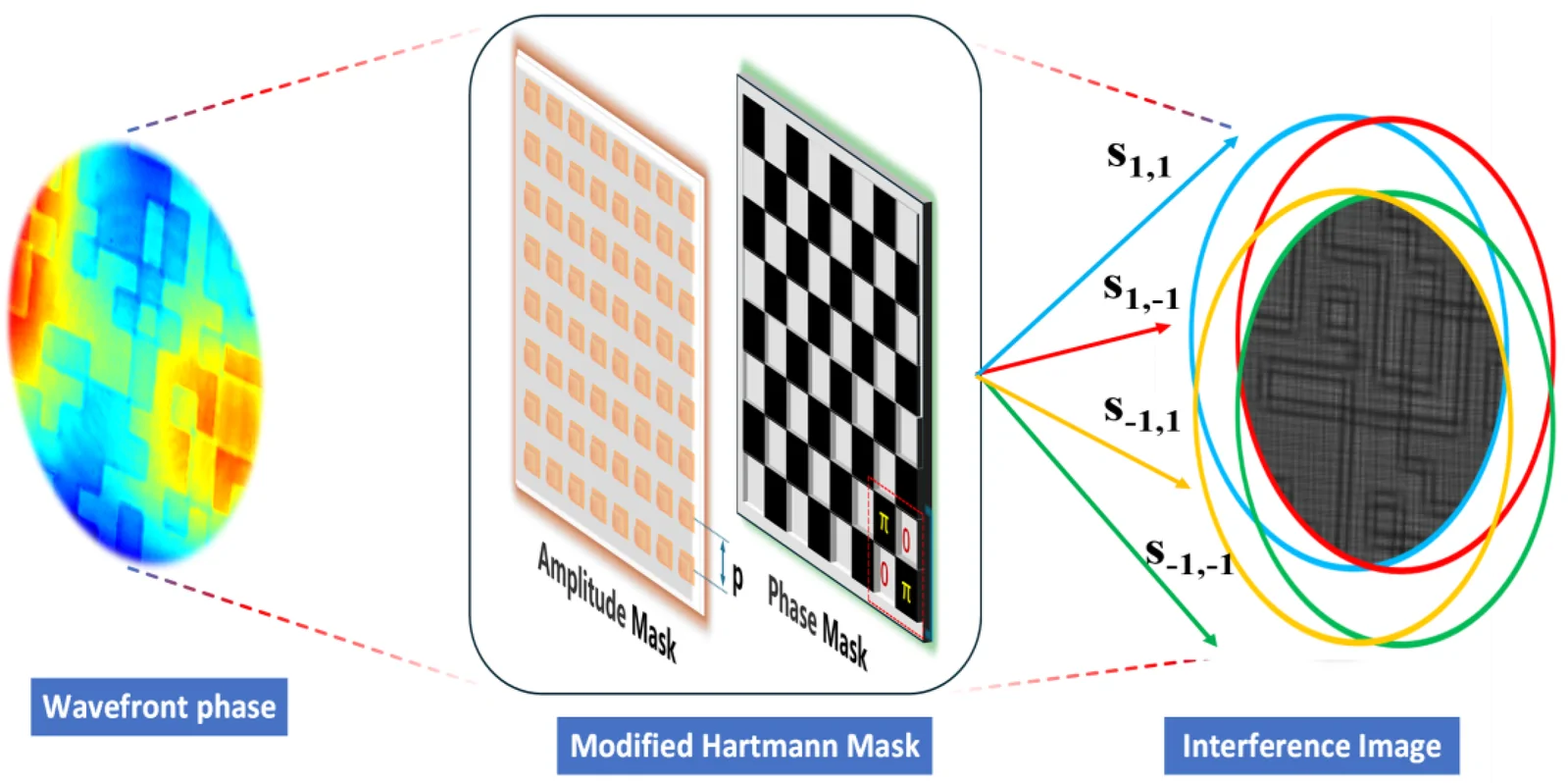
Schematic of the quadri-wave lateral shearing interferometry (QWLSI) wavefront imaging model and its operating principle. The incident wavefront is spatially modulated by a modified Hartmann mask through amplitude and phase modulation, generating four laterally sheared wavefront replicas that interfere at the detection plane.

Quadri-wave lateral shearing interferometry (QWLSI) is a new type of wavefront sensor which was proposed in 2000 [1], and its principle is shown in Figure 1. A quadri-wave lateral shearing interferometer generates two-dimensional interferograms by replicating and mutually interfering with the wavefront under test after it passes through a specially designed diffractive element—a modified Hartmann mask grating.

An incident monochromatic wavefront U(x,y)=A(x,y)exp[iϕ(x,y)] impinges upon a periodic amplitude or phase grating configured as a modified Hartmann mask. Unlike a conventional Hartmann mask with isolated apertures, the modified grating consists of a two-dimensional periodic array of identical sub-apertures, each functioning as a small diffraction-limited source. Under the paraxial approximation, the field immediately after the grating can be expressed as the product of the incident field and the grating complex amplitude transmittance T(x,y).

The grating is designed to select and transmit only the first diffraction orders along the two orthogonal axes. It induces shears of s in the x and y directions, while the zero-order and higher harmonics are suppressed. After propagation over a distance z corresponding to the Talbot or fractional Talbot regime, self-imaging conditions prevail. Four replicas of the original wavefront $U_{+x,+y},U_{+x,-y},U_{-x,+y},U_{-x,-y}$ then emerge, each carrying a mutual lateral displacement. These four copies overlap spatially on the detection plane (e.g., an image sensor). The resulting intensity distribution records their interference:

(1) $$I(r)={\left|\sum_{m=\pm 1}\sum_{n=\pm 1}U(r-s_{m,n})\right|}^{2}$$

where $s_{m,n}=(ms,ns)$ denotes the shear vector for diffraction order (m,n).

Over the past decade, QWLSI has been commonly used in applications such as large-aperture optical surface measurement [2,3], laser beam evaluation [4,5], biological sample imaging [6,7], and metasurface measurement [8,9,10], among others.

**Figure 2:**
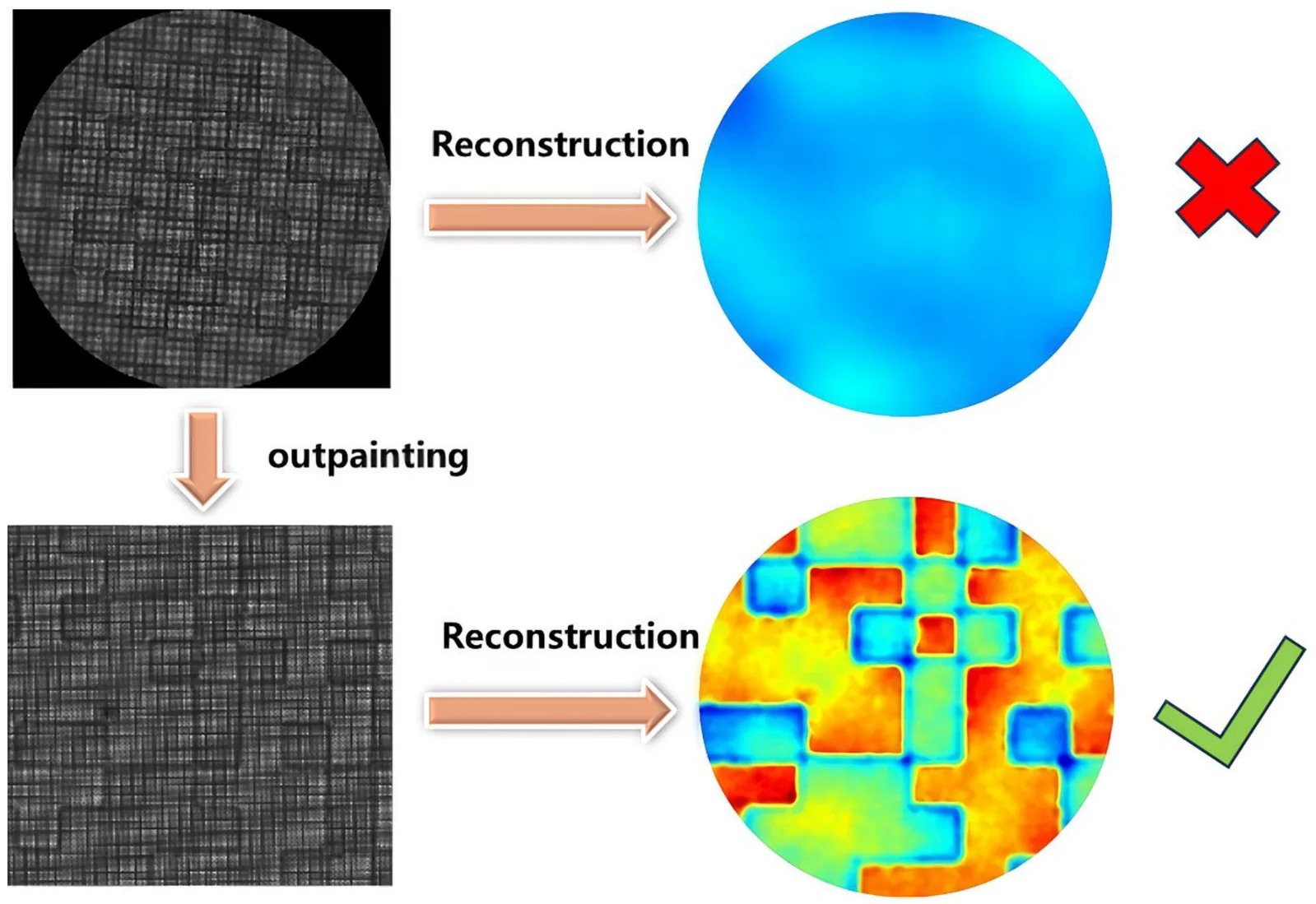
Schematic illustration of the effect of circular-pupil truncation and the proposed QW-GAN-based interferogram outpainting for wavefront reconstruction. Direct reconstruction from the truncated interferogram produces an erroneous wavefront due to the loss of spatial information, whereas QW-GAN-based outpainting restores the missing interferogram regions and enables accurate wavefront reconstruction.

However, in quantitative phase imaging [11,12] and wavefront reconstruction [13,14], interferograms are typically truncated by a circular aperture, resulting in the loss of crucial information near the pupil boundary. This missing boundary information can lead to inaccuracies during the reconstruction process. Moreover, truncation at the pupil boundary causes spectral leakage, which, when directly reconstructed in the frequency domain, can introduce significant errors, as illustrated in Figure 2.

Traditional image outpainting methods are generally classified into two categories: diffusion-based [15,16,17] and patch-based approaches [18,19,20]. However, these methods are typically slow due to their complex iterative processes. In addition, the models often generate errors, causing the outpainted regions to lack consistency with the texture of the original image.

In this paper, we propose a high-performance method for outpainting quadri-wave lateral shearing interferograms based on the Quadri-Wave Generative Adversarial Network (QW-GAN). The reconstruction accuracy is analyzed and compared for defocus, astigmatism, coma, spherical aberration, and random aberrations.

Interferogram truncation caused by the finite circular aperture of the detector is a frequent and practically relevant situation in quadri-wave lateral shearing interferometry (QWLSI): without the missing fringe information, frequency-domain wavefront reconstruction suffers from spectral leakage, spectrum broadening, and Gibbs-type boundary artifacts that bias the retrieved phase. Restoring the truncated fringe regions before Fourier-domain processing is therefore essential for accurate quantitative phase measurement, and this demand for reliable single-shot restoration is precisely what motivates the learning-based approach developed in this work.

The main contributions of this work are as follows: (1) a Generative Adversarial Network (GAN) architecture specifically designed for the outpainting of quadri-wave lateral shearing interferograms is proposed; (2) the proposed method effectively restores the missing regions of circularly truncated interferograms; and (3) the reconstructed interferograms are incorporated into the frequency-domain wavefront reconstruction process, resulting in a 74% reduction in wavefront reconstruction error compared with direct reconstruction, as validated by simulation and experimental results.

Compared with recent deep learning schemes that recover the wavefront phase directly from truncated interferograms through a single convolutional network [21,22,23], the present approach addresses the problem from a complementary and, in our view, more transparent perspective: instead of mapping incomplete interferograms directly to phase, we first restore the missing fringe information in the spatial domain and then recover the phase with the conventional frequency-domain algorithm. This two-stage design keeps the physics-based reconstruction chain (carrier-frequency extraction, Fourier-domain filtering, and phase unwrapping) fully intact and interpretable, while confining the learned component to a well-posed image-to-image regression task. It is this modularity that distinguishes the proposed QW-GAN-based outpainting from end-to-end alternatives and constitutes the main novelty of the work: the network does not compete with the physical forward model; it supplies the frequency analysis with complete input data, thereby eliminating the truncation-induced artifacts at their source.

## 2. QW-GAN Model and Frequency-Domain Phase Reconstruction Method

The proposed framework consists of two major stages: interferogram completion and frequency-domain phase reconstruction.

**Figure 3:**
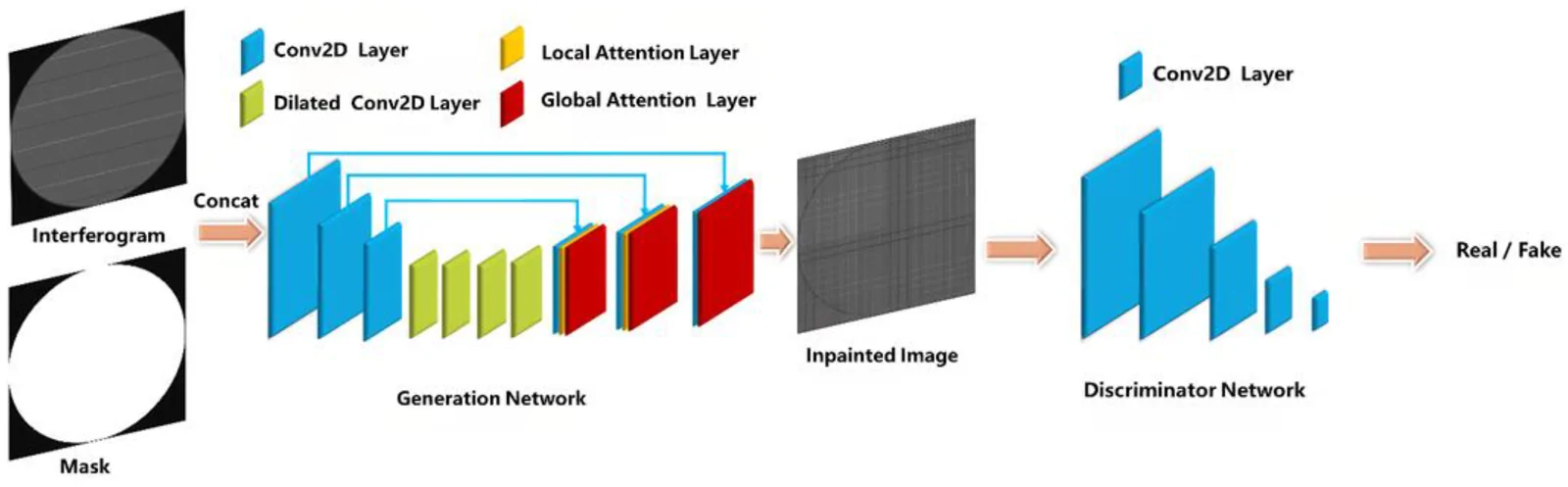
Architecture of the proposed QW-GAN and the interferogram outpainting process. The interferogram and its corresponding mask are first fed into the generator, where Conv2D, dilated Conv2D, local attention, and global attention modules are employed for hierarchical feature extraction and fusion to generate the completed interferogram. The generated and real interferograms are subsequently fed into the discriminator for real/fake classification, and adversarial training is employed to improve the accuracy and structural consistency of the restored missing regions.

In the first stage, a QW-GAN-based outpainting network is developed to recover the missing boundary information caused by circular-aperture truncation, thereby restoring a complete interferogram with preserved fringe characteristics and phase-related features. The proposed QW-GAN outpainting network is designed to perform interferogram outpainting for missing regions, with the input images having a size of 512 × 512 pixels, as demonstrated in Figure 3. The network architecture is flexible and can be adjusted according to the size of the input image. The architecture consists of two primary components—the generator network and the discriminator network—working in an adversarial manner to improve the quality of the reconstructed interferograms.

The generator consists of several 2D convolutional and dilated convolutional layers. The 2D convolutional layers extract local features from the interferograms, such as fringe patterns and intensity variations. Dilated convolutions enlarge the receptive field without substantially increasing the number of network parameters, allowing the generator to use information from a wider surrounding area when reconstructing the missing regions. This is particularly useful for interferograms, where the fringe structure in the missing region is related to the surrounding fringe patterns.

Local and global attention mechanisms are further introduced to improve the reconstruction of the missing regions. Local attention helps the network identify and preserve fine fringe details near the completion boundary, while global attention captures relationships between distant image regions and helps maintain the overall fringe structure. By combining local feature extraction with broader spatial information, these modules enable the generator to produce missing regions that are more consistent with the observed interferogram and reduce discontinuities and unwanted artifacts in the outpainted results.

It is worth clarifying the input–output setting of the generator, since it differs from the unconditional image-generation paradigm in which a generator maps white noise to synthetic images. Here, the generator is conditioned on the truncated interferogram itself and is trained to regress the corresponding complete interferogram, i.e., an image-to-image task; the discriminator then distinguishes the outpainted result from the ground-truth complete interferogram. The training data are therefore used as conditional inputs of the generator, which is the standard conditional-GAN (cGAN) configuration and not a patient data-reproduction mechanism; this design guarantees that the restored fringe structures remain physically consistent with the measured fringe pattern.

The discriminator network consists of several 2D convolutional layers, which are responsible for evaluating the quality of the generated interferogram. The discriminator’s goal is to distinguish between real and generated (outpainted) regions, ensuring that the generated output is as close as possible to the original image in terms of texture and structure. The adversarial loss function between the generator and discriminator ensures that the generator learns to produce realistic and high-quality interferograms. To ensure both the accuracy and authenticity of the outpainted interferograms, the model is trained to minimize both the pixel-wise $L_{1}$ loss (instead of mean square error) and the WGAN adversarial loss $L_{WG}$, which is expressed as

(2) $$L_{G}=L_{1}+\lambda L_{WG}$$

where λ is a coefficient that balances the loss function.

A pixel-wise loss is employed to constrain the generated interferograms to remain consistent with the available spatial information and the corresponding ground-truth data, which is particularly important for preserving the intensity distribution and interference-fringe phase structure required for subsequent wavefront reconstruction. However, relying solely on a pixel-wise loss may favor an averaged solution and consequently produce over-smoothed fringe patterns, especially in the missing regions. Therefore, the WGAN adversarial loss is introduced to provide an additional distribution-level constraint, encouraging the generated interferograms to exhibit statistically and structurally plausible fringe characteristics similar to those of the ground-truth interferograms. The combination of the two losses thus provides complementary constraints: the pixel-wise loss maintains data fidelity, whereas the adversarial loss promotes structural realism. This design is expected to improve the continuity and physical plausibility of the outpainted interferograms and, consequently, reduce errors propagated to the subsequent frequency-domain wavefront reconstruction.

In Figure 3, the generator receives the 512 × 512 truncated interferogram as input; the encoder, built from 2D convolutions and dilated convolutions, extracts multi-scale spatial features; the attention blocks perform local and global feature re-weighting to capture both fine fringe texture and long-range periodic structure; and the decoder progressively up-samples the features, fusing them with encoder features through skip connections, to produce the outpainted complete interferogram. The discriminator branch then evaluates the realism of the generated image against the ground-truth complete interferogram and feeds the adversarial gradient back to the generator during training.

The training process utilizes a dataset composed of simulated and experimental aberration wavefront interferograms (defocus, astigmatism, coma, spherical, etc.), which represent typical distortions in optical systems. These training samples provide the model with diverse and realistic data, enabling it to generalize well to various interferogram reconstruction tasks.

**Figure 4:**
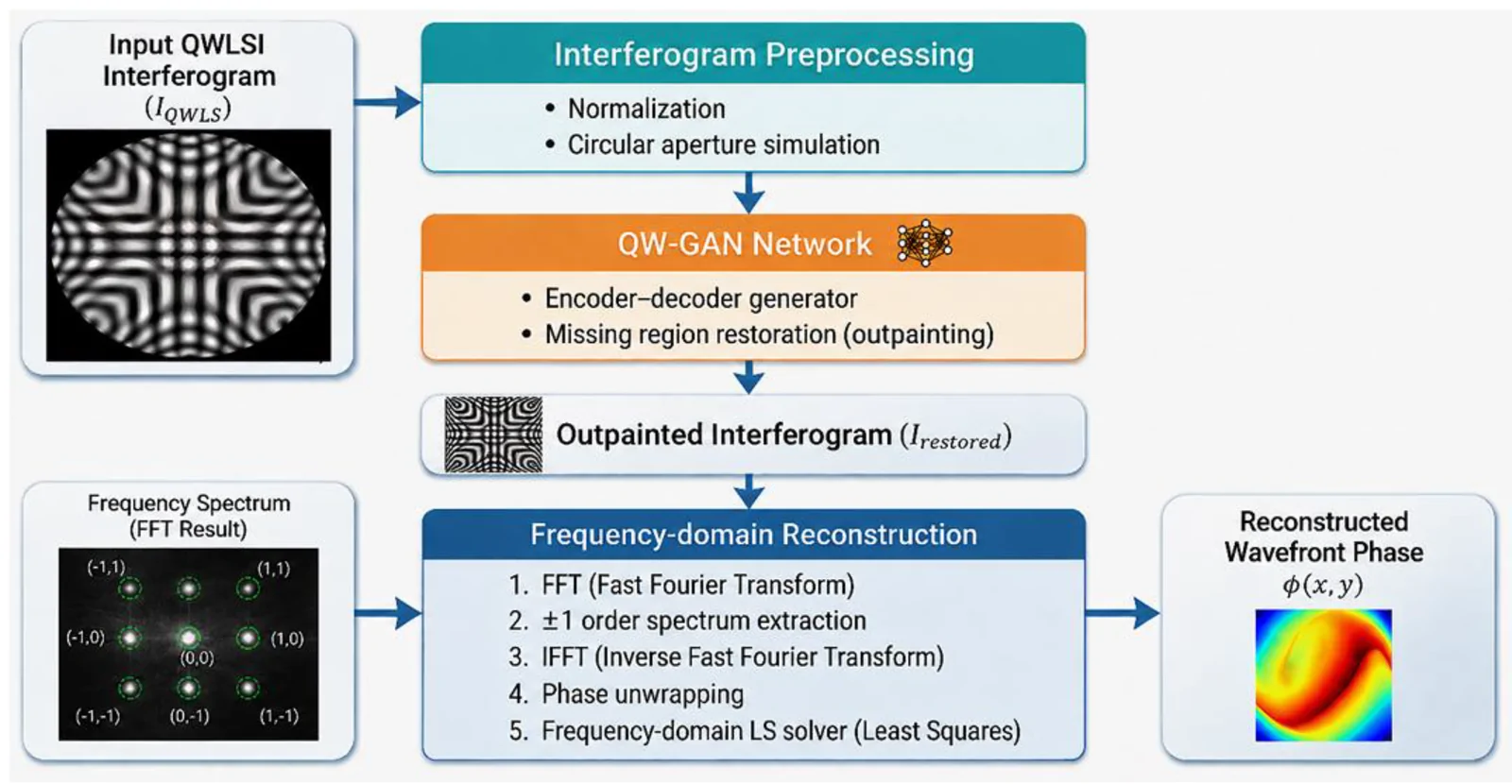
Schematic diagram of the proposed QW-GAN-based interferogram outpainting and frequency-domain phase reconstruction method.

By eliminating truncation-induced discontinuities, the proposed approach effectively reduces spectral leakage and Gibbs artifacts during subsequent frequency-domain analysis in the second stage, as illustrated in Figure 4.

The outpainted interferogram is first used to reduce the discontinuity between the observed and missing regions, thereby mitigating the spectral leakage and Gibbs artifacts associated with circular-aperture truncation. The subsequent frequency-domain wavefront reconstruction consists of the following steps: First, a two-dimensional fast Fourier transform (2D-FFT) is applied to the restored interferogram to obtain its spatial-frequency spectrum. Second, the first-order diffraction components corresponding to the lateral shearing directions are identified and isolated using spectral filtering, while the zero-order and unwanted spectral components are suppressed. Third, the filtered first-order spectra are shifted to the frequency origin and processed to obtain the phase-gradient information in the corresponding horizontal and vertical shearing directions. Fourth, the wrapped phase-gradient distributions are subjected to phase unwrapping to recover continuous phase information. Finally, the unwrapped phase gradients are integrated using a frequency-domain least-squares algorithm, and the reconstructed wavefront is obtained in the spatial domain through inverse Fourier transformation. In this way, the proposed framework combines deep learning-based interferogram outpainting with the conventional frequency-domain QWLSI reconstruction procedure, enabling wavefront recovery from interferograms acquired under incomplete-aperture conditions.

Compared with conventional iterative phase-retrieval methods such as the Gerchberg–Saxton (GS) algorithm, the proposed QW-GAN framework performs interferogram outpainting through a single forward inference after network training, without requiring repeated propagation and amplitude replacement between the spatial and Fourier domains. In the present implementation, the restoration time is reduced from approximately 45 s using the iterative GS-based approach to approximately 2 s with QW-GAN, corresponding to a 22.5-fold reduction in processing time. This improvement results from replacing the repeated iterative optimization with a fixed forward inference, while maintaining comparable or improved wavefront reconstruction accuracy.

The two approaches also differ in their treatment of incomplete-aperture information. In the GS-based approach, the missing information is recovered indirectly through repeated enforcement of the measured-aperture constraints and propagation-domain constraints, making the reconstruction dependent on the iteration process and constraint conditions. In contrast, QW-GAN learns a direct mapping from circularly truncated interferograms to their complete counterparts and restores the missing regions before frequency-domain wavefront reconstruction. This restoration reduces the discontinuity at the circular-pupil boundary and consequently mitigates the spectral leakage and Gibbs ringing introduced by aperture truncation. Thus, the main advantage of QW-GAN is not simply a reduction in the number of iterations, but the use of a learned prior to reconstruct the missing interferogram information before the subsequent phase reconstruction procedure.

## 3. Simulation and Experimental Results

To evaluate the effectiveness of the proposed QW-GAN-based outpainting reconstruction method, simulated wavefront data were used. The simulation experiment is performed using an Intel(R) Core(TM) i9-12900H (2.9 GHz, 32 GB RAM) and MATLAB software (version R2019b, Math Works, Natick, MA, USA). The deep learning framework used is PyTorch 2.3.0.

A simulated dataset was generated to train the proposed QW-GAN using a supervised learning strategy. Each training pair consists of a complete QWLSI interferogram and its corresponding truncated counterpart. The complete interferograms were generated from randomly synthesized wavefront aberrations using the forward model of QWLSI and served as ground-truth references. To simulate the information loss caused by the finite circular aperture of the detector, a circular-pupil mask was applied to the complete interferograms, resulting in four corner regions without valid interferometric information. These truncated interferograms were used as network inputs, while the original complete interferograms were used as reconstruction targets.

The forward-model-based simulated dataset was split into training, validation, and test subsets; in total, 80,000 complete/truncated interferogram pairs were used for training, 10,000 pairs for validation, and 10,000 pairs for testing, with the aberration coefficients of the test set drawn from distributions not seen during training in order to assess generalization. The network was optimized with the Adam optimizer (learning rate 1 × 10^−4^, batch size 16) for 200 epochs on a single NVIDIA GPU (3070Ti laptop), with a total training time of approximately 24 h.

During training, QW-GAN learns the nonlinear mapping between incomplete and complete interferometric patterns, enabling the generator to recover missing fringe structures while preserving the global phase-related characteristics of the interferogram. Through extensive training with diverse aberration distributions, the network establishes the implicit relationship between fringe morphology and wavefront information, providing accurate interferogram restoration for subsequent frequency-domain wavefront reconstruction.

Here, the term forward-model-based simulation is used in the following precise sense: the training interferograms were generated by numerically propagating randomly synthesized wavefront aberrations through the QWLSI forward model, i.e., the same optical model that describes the experimental sensor. The only quantity that is not directly measured is the wavefront itself; the interferogram formation through the quadri-wave lateral shearing principle, the finite-aperture truncation, and the detector sampling all follow the physical transfer function of the instrument. The simulation is therefore physics-based rather than purely statistical, which is why the network trained on this dataset transfers to the experimental measurements presented below.

**Figure 5:**
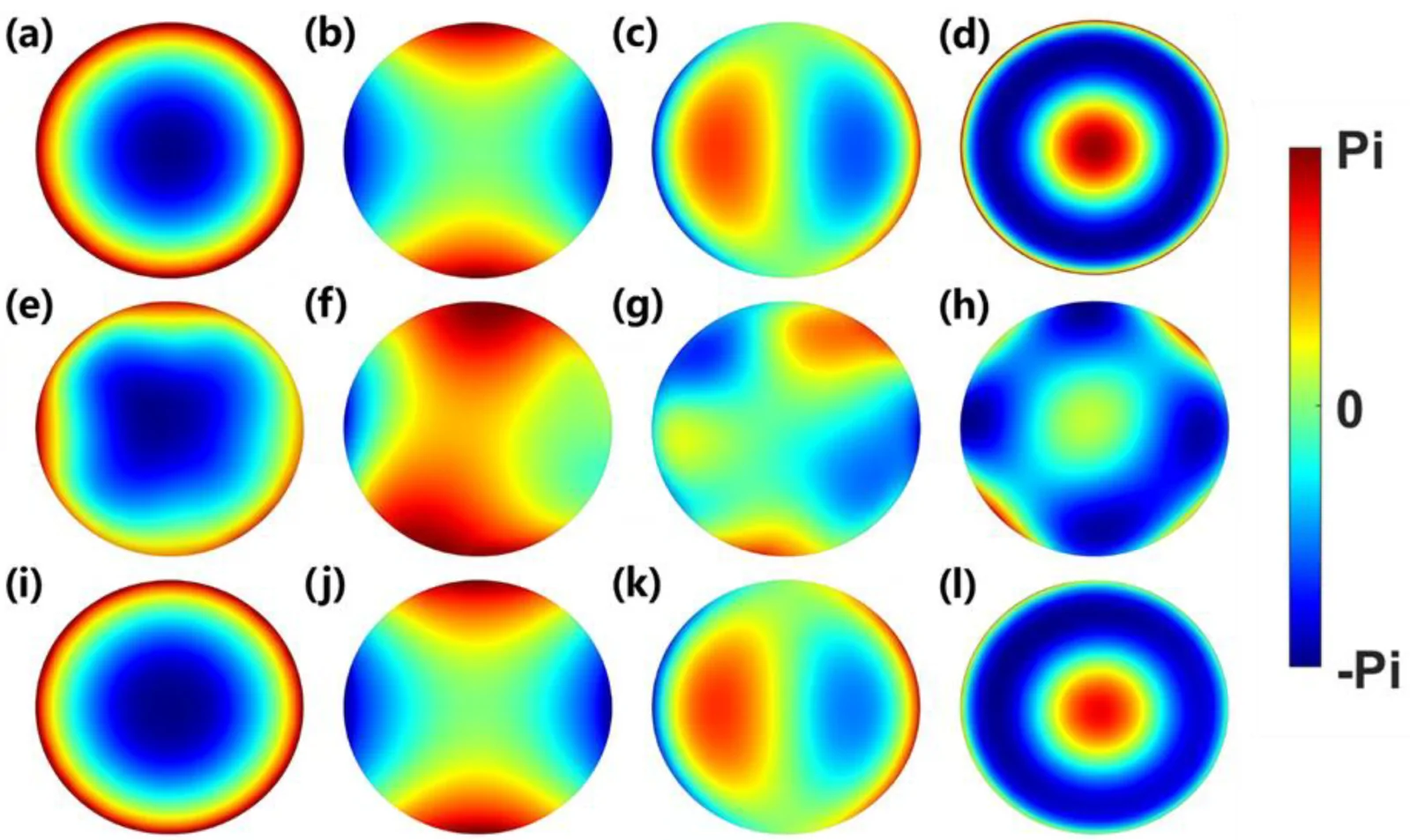
Comparison of the simulated original wavefront phases and the reconstructed results with and without interferogram outpainting. (**a**–**d**) Original wavefront phases corresponding to defocus, astigmatism, coma, and spherical aberration, respectively. (**e**–**h**) Reconstructed wavefront phases obtained directly from the corresponding interferograms truncated by a circular pupil without outpainting. (**i**–**l**) Reconstructed wavefront phases obtained using the corresponding interferograms completed by the proposed QW-GAN network.

Figure 5 presents the simulation comparison of wavefront reconstruction before and after QW-GAN-based interferogram outpainting for four representative aberrations, including defocus, astigmatism, coma, and spherical aberration. The first row (a)–(d) shows the ground-truth wavefront phases, the second row (e)–(h) shows the reconstruction results obtained directly from the truncated interferograms without outpainting, and the third row (i)–(l) presents the reconstructed phases after QW-GAN restoration. As can be observed, direct reconstruction from incomplete interferograms introduces significant phase distortions and structural errors due to spectral leakage caused by the circular aperture. After applying the proposed QW-GAN outpainting method, the reconstructed wavefronts exhibit excellent agreement with the ground truth for all aberration types, with the overall phase distributions and characteristic features being accurately recovered. These results demonstrate that the proposed method effectively compensates for missing interferogram information, suppresses reconstruction artifacts, and significantly improves the accuracy and robustness of frequency-domain wavefront reconstruction.

A quantitative comparison of the wavefront reconstruction accuracy between the proposed method and direct reconstruction is provided in Table 1.

**Figure 6:**
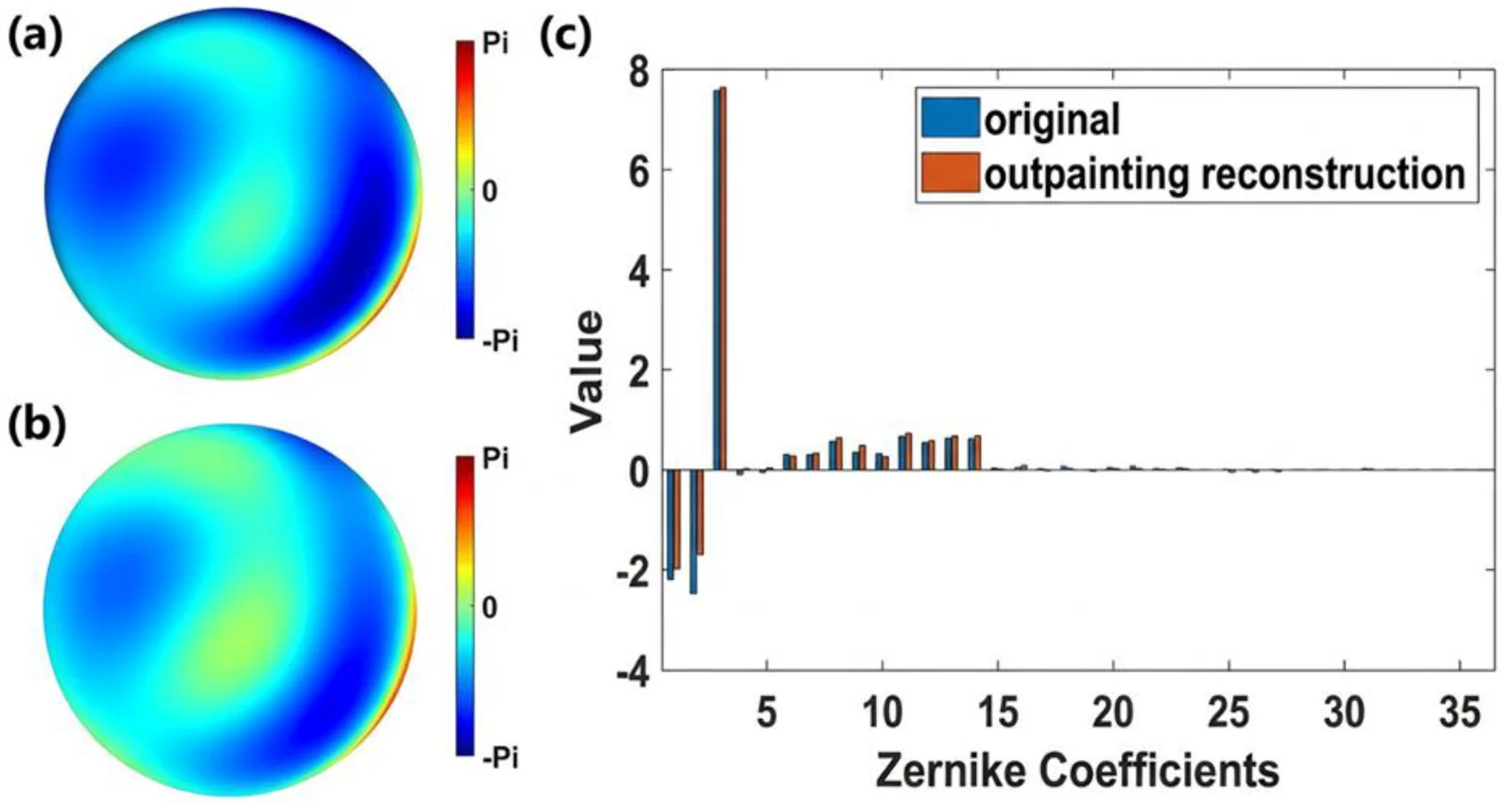
(**a**) Simulated random phase; (**b**) reconstruction phase based on QW-GAN outpainting algorithm; (**c**) comparison of Zernike aberration coefficients between the original and the outpainting-reconstructed wavefront.

Figure 6 provides a quantitative evaluation of the proposed QW-GAN-based interferogram outpainting method using Zernike aberration coefficients. The left panels show the randomly generated wavefront phase and the corresponding reconstructed phase obtained after QW-GAN restoration, while the right panel compares their Zernike coefficients. It can be observed that the reconstructed coefficients agree well with the original simulated values over the entire Zernike spectrum, with nearly identical magnitudes and variation trends, particularly for the dominant low-order aberration terms. These results demonstrate that the proposed outpainting method effectively recovers the missing interferometric information and preserves the original aberration characteristics, thereby ensuring accurate wavefront phase reconstruction after frequency-domain processing.

**Figure 7:**
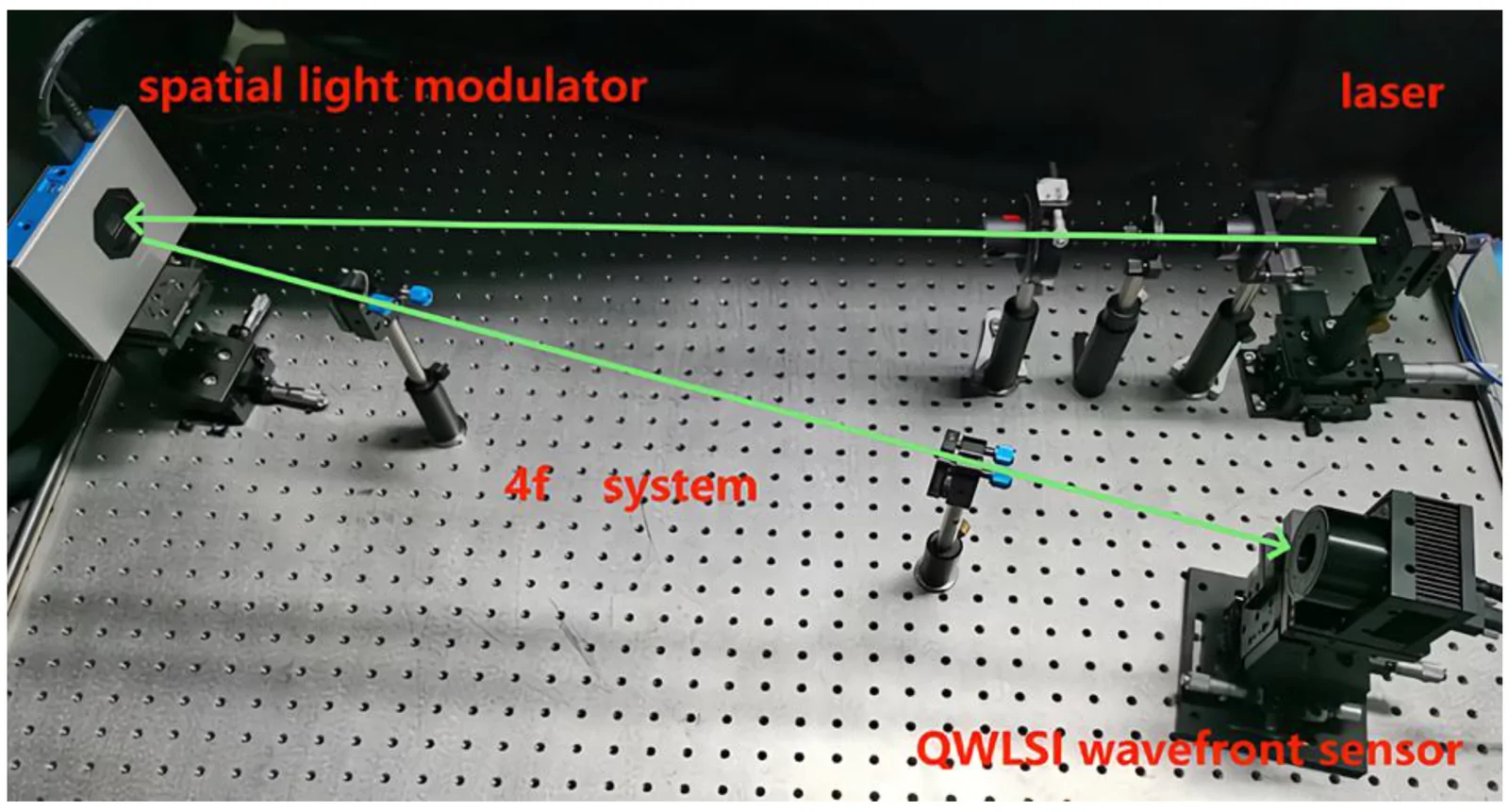
Real experimental setup of quadri-wave lateral shearing interferometry (QWLSI).

To experimentally validate the proposed QW-GAN-based interferogram outpainting method, a QWLSI wavefront measurement system was constructed, as shown in Figure 7. A 532 nm continuous-wave (CW) laser was used as the illumination source. After passing through a circular aperture, the expanded and spatially filtered laser beam was incident on a spatial light modulator (SLM), where predefined phase distributions were loaded to generate various test wavefronts. The SLM was employed to emulate different optical aberrations by modulating the phase of the incident wavefront, enabling flexible generation of phase patterns with known characteristics.

For completeness, the key specifications of the experimental components are stated here. Spatial light modulator: a phase-only reflective SLM with a resolution of 1920 × 1200 pixels and a pixel pitch of 8 µm, providing up to 2π (6.28) rad of phase modulation at the operating wavelength of 532 nm with 8-bit gray levels and a refresh rate of 60 Hz. The QWLSI sensor employed in the experiment was a modified Hartmann mask sensor, the specifications of which are listed in Table 2.

Regarding the non-ideal response of the SLM, three practical issues were addressed in the experiments. First, phase-only SLMs exhibit a nonlinear voltage-to-phase response; a lookup-table calibration was performed prior to the measurements so that the loaded patterns matched the intended aberration maps. Second, the SLM was operated in a static mode during each single-shot acquisition, so that frame-to-frame crosstalk (flicker) did not corrupt the captured interferograms, consistent with the single-shot measurement scenario targeted by the proposed method. Third, the SLM and the camera were synchronized through hardware trigger signals, and the camera frame rate was set sufficiently below the SLM refresh rate to guarantee that every recorded frame corresponded to a fully settled SLM pattern. With these measures, the experimental interferograms deviate only mildly from the ideal forward-model simulations upon which the QW-GAN was trained.

The modulated wavefront was subsequently transmitted through a 4f optical imaging system, which was used to relay the SLM plane onto the QWLSI wavefront sensor plane while maintaining the spatial phase information. The 4f system also ensured accurate spatial filtering and magnification control during wavefront propagation. The output wavefront from the 4f system was then captured by the QWLSI sensor, where the incoming wavefront was converted into a quadri-wave lateral shearing interferogram through the microoptical shear element. The generated interferograms were recorded by the detector for subsequent processing and wavefront reconstruction.

**Figure 8:**
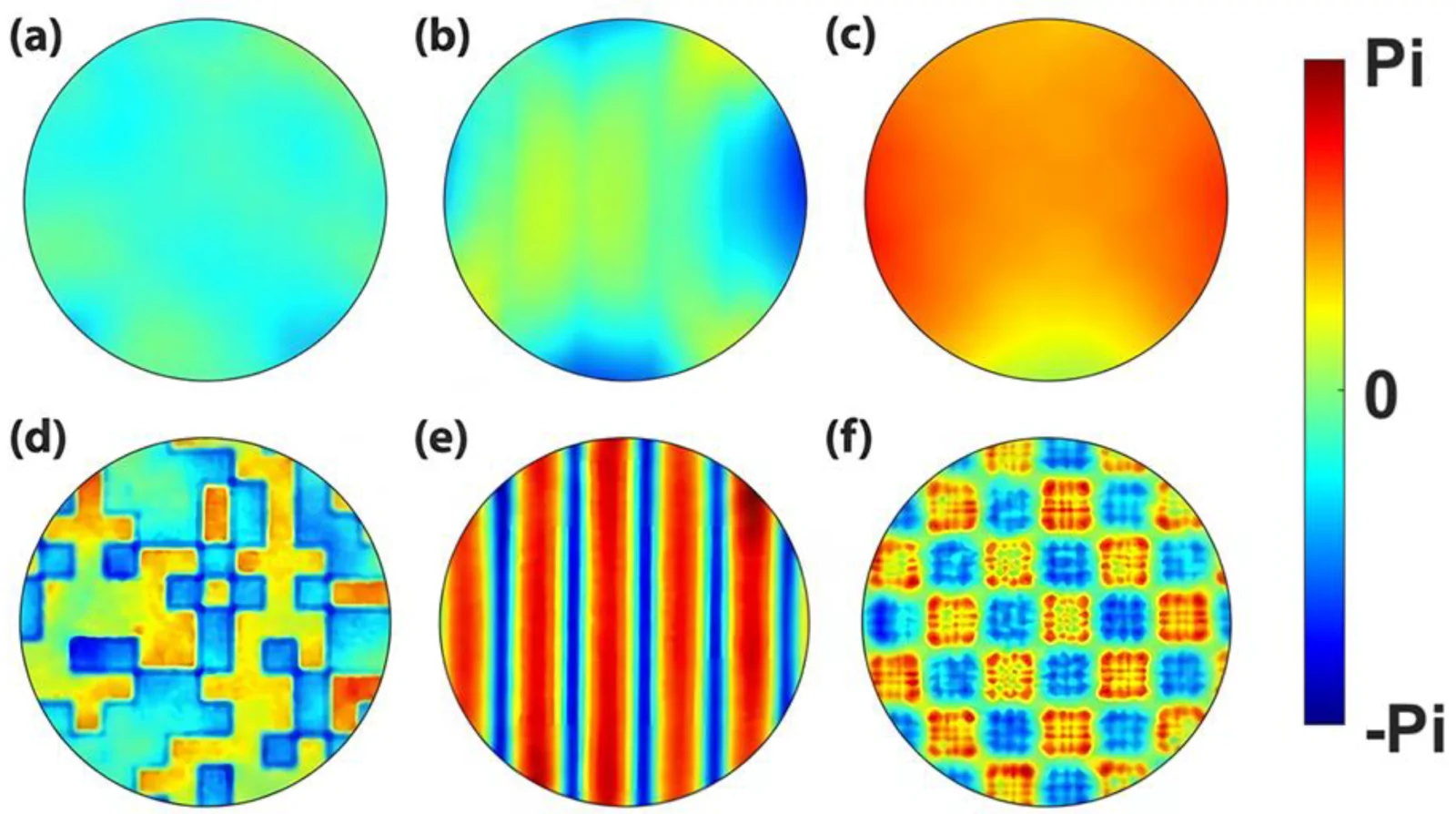
Real experiment wavefront reconstruction: (**a**–**c**) without outpainting; (**d**–**f**) with QW-GAN outpainting: (**d**) random step phase; (**e**) sinusoidal fringes; (**f**) checkerboard phase.

In the experiment, different phase patterns, including random phase distributions, sinusoidal fringes and a checkerboard phase, were sequentially loaded onto the SLM to generate corresponding QWLSI interferograms. The acquired experimental interferograms were further processed using the proposed QW-GAN network to restore the missing regions caused by aperture truncation, and the reconstructed interferograms were subsequently used for frequency-domain wavefront phase reconstruction in Figure 8. This experimental configuration provides a controllable platform for evaluating the effectiveness and robustness of the proposed interferogram restoration method under realistic measurement conditions.

## 4. Discussion

Reconstruction results. Figure 5 shows that the improvement is most evident for defocus, astigmatism, coma, and spherical aberration. For these low-order aberrations, the missing corner regions contain substantial fringe information, and direct reconstruction produces noticeable phase errors near the boundary. After QW-GAN outpainting, the reconstructed phase approaches that obtained in the central region. For high-order aberration, the overall improvement is smaller, but the boundary errors are also reduced. The main reason is that the restored fringe field preserves the local fringe orientation and spatial period inferred from the measured region. This provides a more appropriate input for subsequent Fourier-domain filtering and phase extraction. The reconstruction performance therefore depends on both the quality of the restored interferogram and the accuracy of the extracted first-order spectral components.

Robustness. The reliability of QW-GAN naturally degrades when the truncated region becomes very large, because the extrapolation then relies on an increasingly small amount of intact context. In the extreme case in which almost the entire interferogram is missing, the outpainted fringe pattern is dominated by the learned prior and may no longer match the actual measurement; the subsequent frequency-domain reconstruction would then inherit this uncertainty. For the truncation geometry addressed in this work (four corner regions removed by a circular detector aperture), we recommend restricting the method to comparable or milder truncation ratios.

Practical considerations. Several practical issues should be considered. Moderate sensor noise has limited influence on the restoration results in the present simulations. The L1 reconstruction term provides relatively stable pixel-level recovery, while the adversarial constraint helps preserve the spatial characteristics of the fringe pattern. For computation, the generator requires only a few milliseconds for a single forward pass on the reference GPU. The Fourier filtering, phase extraction, and phase unwrapping stages are currently performed on the CPU and constitute the main part of the processing time. GPU implementation of these operations would therefore be necessary for a fully real-time system. The restored boundaries did not show noticeable over-smoothing in the experiments. Nevertheless, fine fringe structures or localized defects may be attenuated in some cases. Additional structural or frequency-domain constraints could improve the preservation of such features. Although the present method was developed for QWLSI, the same strategy can be extended to other interferometric measurements affected by aperture truncation, provided that the corresponding fringe-generation model is used to construct the training data.

Quantitative comparison. The quantitative results in Table 1 show a consistent improvement in the outpainting-based reconstruction over direct reconstruction for all four aberration types. The reduction in error is particularly apparent near the aperture boundary, where the influence of truncation is strongest. The result is consistent with the reduction in boundary discontinuities observed in the restored interferograms. In terms of computational efficiency, the proposed method requires only a single generator inference during reconstruction, avoiding the repeated iterations required by conventional iterative phase-retrieval approaches. This provides an advantage for high-throughput wavefront measurements. Further optimization of the Fourier-domain processing and phase unwrapping stages is required before the complete pipeline can be used in real-time closed-loop applications.

Overall assessment. The results indicate that interferogram outpainting can effectively compensate for the information loss caused by circular-aperture truncation in QWLSI. Its main advantage is that the conventional frequency-domain reconstruction framework is retained, while the missing fringe information is restored before phase extraction. The method performs reliably for the truncation geometry investigated here, but its accuracy depends on the availability of sufficient measured context and on the representativeness of the training data. Future work will focus on larger and more irregular missing regions, stronger spatial-frequency consistency constraints, and GPU implementation of the complete reconstruction pipeline. These developments are expected to further improve the applicability of the method to high-speed and closed-loop wavefront sensing.

## 5. Conclusions

In summary, this work established that spatial-domain restoration of aperture-truncated QWLSI interferograms is a sound alternative to end-to-end phase regression: by supplying the conventional frequency-domain analysis with complete fringe data, the proposed QW-GAN eliminates spectral leakage, spectrum broadening, and Gibbs ringing at their source while preserving the interpretability of the processing chain. Both the forward-model-based simulations and the experiments on a phase-only SLM consistently show reduced reconstruction error, especially near the pupil boundary, at a computational cost compatible with real-time wavefront sensing.

Several directions merit further investigation: extending the method to more severe truncation geometries and partial-occlusion cases; incorporating frequency-domain consistency and structural-similarity terms to further reduce residual boundary artifacts; and porting the complete pipeline from carrier extraction to phase unwrapping to the GPU to enable closed-loop adaptive optics deployment. We believe the demonstrated combination of learning-based restoration and physics-based reconstruction will also benefit related single-shot wavefront sensing modalities beyond QWLSI.

## Figures and Tables

**Table 1:** Quantitative comparison of wavefront reconstruction accuracy (averaged over defocus, astigmatism, coma, and spherical aberration in the simulation).

Metric	Direct (Truncated)	QW-GAN Outpainting
RMS (λ)	0.077	0.020
SSIM	0.7117	0.9795
Correlation coefficient	0.8112	0.9969

**Table 2:** Specifications of QWLSI sensor.

Parameter	Value
Spectral Response Range	400–1100 nm
Sensor Array Resolution	2048 × 2048 pixels
Pixel Pitch	6.5 um
Grating Pitch	26 um
Phase Sampling	512 × 512
Real-Time Processing Frequency	1 Hz
Interface	Ethernet

## Data Availability

All data can be provided upon reasonable request to the corresponding author.

## Abbreviations

The following abbreviations are used in this manuscript:

QWLSI	Quadri-Wave Lateral Shearing Interferometry
GAN	Generative Adversarial Networks
